# Critical closing pressure as a new hemodynamic marker of cerebral small vessel diseases burden

**DOI:** 10.3389/fneur.2023.1091075

**Published:** 2023-03-21

**Authors:** Xian Fu, Weijin Zhang, Xianliang Li, Hongying Liu, Yin Zhang, Qingchun Gao

**Affiliations:** ^1^Department of Neurology, Shenzhen Bao'an District Songgang People's Hospital, Shenzhen, China; ^2^Department of Neurology, Institute of Neuroscience, The Second Affiliated Hospital of Guangzhou Medical University, Guangzhou, China

**Keywords:** critical closing pressure, cerebral small vessel disease burden, cerebrovascular tone, cerebrovascular hemodynamics, transcranial Doppler

## Abstract

**Purpose:**

To investigate cerebrovascular hemodynamics, including critical closing pressure (CrCP) and pulsatility index (PI), and their independent relationship with cerebral small vessel disease (CSVD) burden in patients with small-vessel occlusion (SVO).

**Methods:**

We recruited consecutive patients with SVO of acute cerebral infarction who underwent brain magnetic resonance imaging (MRI), transcranial Doppler (TCD) and CrCP during admission. Cerebrovascular hemodynamics were assessed using TCD. We used the CSVD score to rate the total MRI burden of CSVD. Multiple regression analysis was used to determine parameters related to CSVD burden or CrCP.

**Results:**

Ninety-seven of 120 patients (mean age, 64.51 ± 9.99 years; 76% male) completed the full evaluations in this study. We observed that CrCP was an independent determinant of CSVD burden in four models [odds ratio, 1.41; 95% confidence interval (CI), 1.17–1.71; *P* < 0.001] and correlated with CSVD burden [β (95% CI): 0.05 (0.04–0.06); *P* < 0.001]. In ROC analysis, CrCP was considered as a predictor of CSVD burden, and AUC was 86.2% (95% CI, 78.6–93.9%; *P* < 0.001). Multiple linear regression analysis showed that CrCP was significantly correlated with age [β (95% CI): 0.27 (0.06 to 0.47); *P* = 0.012], BMI [β (95% CI): 0.61 (0.00–1.22)] and systolic BP [β (95% CI): 0.16 (0.09–0.23); *P* < 0.001].

**Conclusions:**

CrCP representing cerebrovascular tension is an independent determinant and predictor of CSVD burden. It was significantly correlated with age, BMI and systolic blood pressure. These results provide new insights in the mechanism of CSVD development.

## Introduction

Cerebral small vessel disease (CSVD) is the main cause of lacunar stroke, cerebral hemorrhage and dementia in elderly patients (1). It represents various pathophysiological processes that affect the structure or function of cerebral microvessels; however, the underlying pathogenesis of CSVD, which might involve genetic factors and pathophysiological mechanism, is largely unclear (2, 3).

Numerous studies have recently emphasized the important role of cerebrovascular hemodynamics in the pathophysiological mechanism of CSVD, particularly concerning cerebral blood flow (CBF), cerebrovascular reactivity (CVR) and pulsatility index (PI) (4–8). These hemodynamic parameters are used to examine microvascular function and represent current and ongoing possible mechanisms of CSVD. Critical closing pressure (CrCP) firstly introduced by Burton is also a very influential cerebral hemodynamic parameter, which is significantly correlated with CBF and PI (9–13).

CrCP indicates an arterial blood pressure (ABP) threshold. Below this threshold, the local microvascular blood pressure is inadequate to prevent collapse, and the CBF approaches zero (13). According to Burton's model, CrCP is equal to the sum of vascular wall tension and intracranial pressure (ICP) (13, 14). Previous studies have demonstrated that CrCP tends to increase with increases of ICP in head injury patients (11, 15, 16). Nonetheless, the results of Weyland et al. (17) reflect the situation that the influence of small changes of ICP on CrCP is mainly determined by the greater effect of active vessel wall tension. Therefore, CrCP may primarily reflect the behavior of cerebrovascular tone rather than ICP in pathological states, and is widely regarded as one of the most clinically relevant parameters to evaluate the changes of cerebrovascular tone (11, 12, 17–20). This conclusion can also be drawn especially in cerebrovascular diseases with relatively constant ICP.

Aaslid et al. (12) creatively presented a non-invasive method for evaluating CrCP by transcranial Doppler (TCD), which made it possible to measure CrCP non-invasively in patients and be widely applied to clinical studies. Previous clinical studies on CrCP mainly focused on patients with head injury and intracerebral hemorrhage (11, 14–16, 21), and almost did not involve patients with CSVD. Recent studies indicated that CSVD may be related to cerebral hemodynamic function (4, 5, 7). Since CrCP is an important cerebral hemodynamic parameter and index of cerebral vascular tone (9, 11, 17), it is necessary to explore the role of CrCP in the pathogenesis of CSVD.

The total score of CSVD represents the whole burden of CSVD on magnetic resonance imaging (MRI), which provides a more complete estimate of the overall impact of CSVD on the brain (2, 22, 23). Therefore, the aim of the current study was to investigate the determinants of CSVD burden, the relationship between cerebral hemodynamics, including CrCP and PI and CSVD burden, and the correlated factors of CrCP in CSVD patients.

## Methods

### Patients

We collected database for consecutive patients with acute ischemic stroke (AIS) within seven days of symptom onset who were admitted between October 2018 and March 2021. Among these patients, we consecutively selected patients with anterior circulation infarction classified as small vessel occlusion (SVO) according to the Trial of Org 10172 in Acute Stroke Treatment (TOAST) classification system (24). Their National Institutes of Health Stroke Scale (NIHSS) and modified Rankin Scale (mRS) scores were less than three. All participants underwent brain magnetic resonance (MR), TCD and CrCP during admission. We excluded patients who had (1) a history of stroke; (2) arrhythmias that may affect the accurate evaluation of TCD and CrCP; (3) a history of radiotherapy for head and neck cancer; (4) a history of endovascular therapy for anterior circulation diseases; (5) high or medium risk of potential sources of cardiac embolism according to the TOAST classification system; (6) unsuitable temporal windows for conducting TCD and CrCP measurements; (7) Patients with hypercapnia or hypocapnia. The written informed consent for this study was obtained from the patient or his or her family members. The ethics committee of the Second Affiliated Hospital of Guangzhou Medical University approved the study protocol. Good clinical practice guidelines in accordance with the Declaration of Helsinki were used, and patient privacy was strictly protected.

### Data collection

We collected data on sex, age, NIHSS and mRS scores on admission, presence of risk factors [including hypertension, diabetes mellitus, coronary heart disease, current or recent smoking history, alcohol consumption history, body mass index (BMI), triglyceride, cholesterol, low density lipoprotein and glucose], hemodynamic parameters [including blood pressures, pulse pressure, blood flow velocities of middle cerebral artery (MCA), PI and CrCP], CSVD subtype and its score (Table 1). Hypertension was described as present if the subject had been previously diagnosed by a cardiology physician and were routinely receiving antihypertensive therapy. Patients were defined as having type 2 diabetes if they had known diabetes treated by diet, oral hypoglycemic drugs, or insulin before the stroke. Coronary artery disease included any history of heart attack/myocardial infarction, angina, or coronary heart disease. The hemodynamic parameters, such as CBF velocity, PI and CrCP, were measured using TCD. CSVD subtypes and scores were defined according to the standards for reporting vascular changes on neuroimaging (STRIVE) criteria based on the results of the brain MRI (23).

**Table 1:** Demographics and baseline characteristics of participants by CSVD score.

**Characteristics**	**All patients (*n* = 97)**	**CSVD score 1–2 (*n* = 72)**	**CSVD score 3–4 (*n* = 25)**	***P*-value^a^**
Male	74 (76)	53 (74)	21 (84)	0.293
Age, y	64.51 ± 9.99	62.90 ± 9.28	69.12 ± 10.74	0.009
**Vascular risk factors**				
Hypertension	57 (59)	37 (51)	20 (80)	0.012
Diabetes mellitus	15 (16)	11 (15)	4 (16)	0.931
Coronary artery disease	12 (12)	7 (9)	5 (20)	0.179
Current or recent smoking history	28 (29)	22 (31)	6 (24)	0.533
Alcohol consumption history	10 (10)	7 (9)	3 (12)	0.747
BMI	24.47 ± 3.16	24.55 ± 3.22	24.23 ± 3.05	0.658
Triglyceride	1.47 ± 0.87	1.50 ± 0.93	1.37 ± 0.67	0.532
Cholesterol	5.05 ± 1.29	5.12 ± 1.32	4.86 ± 1.21	0.405
Low density lipoprotein	3.38 ± 1.00	3.46 ± 0.99	3.14 ± 0.98	0.168
Glucose, median (IQR)	4.70 (4.26–5.42)	4.69 (4.29–5.54)	4.73 (4.11–5.30)	0.174
**Hemodynamics**				
Systolic blood pressure	135.03 ± 28.25	130.42 ± 22.48	148.32 ± 38.07	0.013
Diastolic blood pressure	77.04 ± 14.80	74.94 ± 11.73	83.08 ± 20.45	0.033
Mean blood pressure	96.37 ± 18.36	93.44 ± 14.27	104.83 ± 25.41	0.016
Pulse pressure	57.99 ± 18.37	55.47 ± 15.95	65.24 ± 22.87	0.031
PSV (cm/s)	91.76 ± 24.30	92.64 ± 25.50	89.24 ± 20.72	0.550
MFV (cm/s)	59.75 ± 15.03	60.46 ± 16.22	57.70 ± 10.99	0.432
PI, median (IQR)	0.84 (0.77–1.01)	0.83 (0.78–1.01)	0.85 (0.77–1.01)	0.961
CrCP	40.44 ± 11.21	37.15 ± 8.25	49.91 ± 13.25	<0.001
**Neuroimaging markers**				
Lacunes	82 (85)	59 (82)	23 (92)	0.231
WMH	50 (52)	28 (39)	22 (88)	<0.001
PVS	56 (58)	34 (47)	22 (88)	<0.001
Microbleeds	18 (19)	2 (3)	16 (64)	<0.001
**CSVD score**				
1	21 (21)	/	/	/
2	51 (53)	/	/	/
3	17 (18)	/	/	/
4	8 (8)	/	/	/

BMI, body mass index; IQR, Interquartile Range; PSV, peak systolic velocity; MFV, mean flow velocity; PI, pulsatility index; CVR, cerebrovascular reactivity; CrCP, critical closing pressure; WMH, white matter hyperintensities; PVS, Perivascular spaces; CSVD, cerebral small vessel disease.

Data presented as mean ± SD, number (%) or median (range).

^a^*p*-value for CSVD score 1–2 vs. score 3–4.

### Brain MRI acquisition and analysis

Brain MRI scans for all participants were acquired through a Siemens 3T MRI system (Erlangen, Germany) employing a 12-channel phased array head coil during admission. The study protocol included axial diffusion-weighted imaging (DWI, TE, 90; TR, 3,200) with b values of 0 and 1,000 s/mm^2^, T2-weighted (TR 7,000; TE 102), fluid-attenuated inversion recovery (FLAIR, TR 7,000; TE 102), gradient echo, and sagittal T1-weighted sequences (TR 511; TE 8.5); FOV, 250 × 250 mm; Mmatrix, 256 × 256. slice thickness was 5 mm with 1 mm gap between slices. The total imaging time was ~45 min for each participant.

All MRIs were independently assessed blinded to clinical information by two experienced neuroradiologists, and their discrepancies were resolved by consensus. Structural image analysis of CSVD features was performed according to the STRIVE criteria (23). Recent small subcortical infarct was defined as a hyperintense area ( ≤ 20 mm) involving subcortical tissue on DWI, with T1-weighted hypointensity, T2-weighted and FLAIR hyperintensity. Lacune of presumed vascular origin was defined as a CSF-filled cavity (3–15 mm) surrounded by a hyperintense rim on FLAIR and T2, and no increased signal on DWI. White matter hyperintensities (WMH) of presumed vascular origin was defined as lesions with hyperintensities on T2-weighted and FLAIR imaging and hypointensities on T1-weighted imaging, and graded according to the modified Fazekas scale (25). We defined perivascular spaces (PVS) as small round (axial) or liner (parallel to vessels) space (<3 mm) with CSF-like signal on all MRI sequences without hyperintense rim on T2-weighted or FLAIR imaging, and they were rated on a semiquantitative scale from 0 to 4. Cerebral microbleeds (CMB) were defined as small (2–10 mm) hypointensity on gradient echo images in cerebellum, brainstem, basal ganglia, white matter, or cortico-subcortical junction. According to previous descriptions, we rated the total MRI burden of CSVD using the CSVD score. Each MRI feature of CSVD was given one point if present, for a maximum score of four.

### Hemodynamic data acquisition and analysis

Hemodynamic parameters were measured by one experienced technician using TCD, continuous blood pressure monitor and capnograph. Participants were in a supine position with their heads slightly elevated after resting for 15 min. TCD basal examination was performed using a commercial machine (DWL Elektronische Systeme GmbH, Sipplingen, Germany) by directing a 2-MHz transducer to the temporal window above each zygomatic arch to detect the blood flow in the middle cerebral artery (MCA). Time-averaged peak systolic velocity (PSV), mean flow velocity (MFV) and PI were generated automatically. Once the signal was optimized, the transducer was locked in place and fixed on the temporal window using a custom fixation device (Marc 600, Spencer probe fixation system, Spencer technologies, USA).

Continuous BP recording was made *via* a tonomatic continuous blood pressure monitor (CBM-7000; Colin corporation, Japan), with the participant's hand maintained at the same level as the head. The measurements were initially corroborated by standard measurements of BP with an automated arm cuff (Omega 1400 series; *In vivo* Laboratories Inc., Orlando, Florida, USA). End-tidal CO_2_ was measured with an automated capnograph (Multi-Box, DWL, Germany). The exhaled air is introduced into the non-invasive capnograph through the double-inserted nasal oxygen tube, and the breathing rhythm and end-tidal CO_2_ concentration were continuously and dynamically recorded. The signals of continuous BP and end-tidal CO_2_ concentration were transmitted to the TCD machine through a dedicated cable, so that the BP, end-tidal CO_2_ concentration curve and MCA blood flow spectrum envelope can be displayed in the monitoring trend window at the same time. After the three waveforms were stable (change rate per minute <10%), the trend graph was continuously recorded for 5 min, and the data were synchronized to the hard disk of the TCD machine.

According to Rune Aaslid's method (12), we used our own offline software to calculate the value of CrCP. We selected the cerebral blood flow wave spectrums with complete envelopes in 6 continuous cardiac cycles (at least greater than one respiratory cycle). To compensate for the time delay between pressure and flow velocity curves at the radial and MCA, flow velocity curves were shifted by an average of 54 msec. The correct compensation of time delay was calculated by iterative regression analysis until hysteresis of BP/FV plots completely disappeared. The least square method was used to analyze the BP/FV relationship line, and the pressure axis intercept of these BP/FV plots represents CrCP of the cerebral circulation. Six continuous cardiac cycles of each measurement period were randomly selected and extrapolated CrCP data of all heart beats within these cardiac cycles were averaged for further analysis.

### Interobserver and intraobserver reliability

Data were analyzed using the interclass correlation (ICC) coefficient, described in detail in the Statistical Analysis section below to determine the interobserver and intraobserver reliability for CSVD score measurment as per all the records measured by two experienced neuroradiologists.

### Statistical analysis

Data are expressed as the mean ± standard deviation (SD) or median (25th and 75th percentiles) for continuous variables and as the frequency and percentage for discrete variables. Comparisons between patients with CSVD scores of different levels were performed by unpaired Student's *t*-test or the Mann–Whitney *U* test where appropriate for continuous variables and the chi-squared test for categorical variables. To evaluate the Determinants of CSVD burden, we performed multivariate logistic regression with adjustments for the variables in four models. To better understand the relationship between CrCP, PI and the CSVD burden, we performed multivariable linear regression analysis, receiver operating characteristic (ROC) curve analysis and established a fractional polynomial plot with 95% confidence intervals (CIs) for the CSVD scores according to the level of CrCP and PI on the basis of the generalized additive regression model. We also performed multivariable linear regression analyses assessing the relationships between CrCP and the baseline characteristics. The ICC coefficient was used as an index of interobserver and intraobserver reliability/agreement. The interobserver and intraobserver reliability were assessed by fitting two-way mixed effects model using the reliability analysis procedure in SPSS, where the value of CSVD score was modeled with neuroradiologist and subject entered as random effects. Statistical significance was established at *P* < 0.05. Statistical analyses were performed using SPSS 17.0 software for Windows (SPSS Inc, Chicago, IL, USA) and Stata 14.0 software for Windows (StataCorp. LP, Texas, USA).

## Results

A total of 120 patients with CSVD were enrolled into this study; 97 participants completed the full hemodynamics evaluation, brain multimodal MR and analyzable data collection. Twenty three patients having unsuitable temporal windows for assessing the important hemodynamics parameters using TCD were excluded. Interobserver and intraobserver reliability using ICC for measuring CSVD score were both 1.0 (*P* < 0.001).

The 97 participants had a mean age of 64.51 ± 9.99 years (range 43–86 years); 74 (76%) were male. Based on the MR imaging features of CSVD, we observed that 82 patients (85%) had lacunes; 50 patients (52%) had WMH; 56 patients (58%) had PVS; and 18 patients (19%) had microbleeds. The CSVD burden was estimated using the total CSVD score. Most patients (53%) scored 2, 21 patients (21%) scored 1, 17 patients (18%) scored 3, and eight patients (8%) scored 4. The values of PI and CrCP were 0.84 (0.77–1.01) and 40.44 ± 11.21 mmHg, respectively. We also observed that patients with higher CSVD score had higher values of CrCP (*p* < 0.01). Demographics and baseline characteristics of participants by CSVD score are shown in Table 1.

### Determinants of CSVD burden

In the univariate analysis (Supplementary Table 1), the factors associated with CSVD burden (*P* < 0.05) were the older age, hypertension, higher blood pressure, pulse pressure, and CrCP levels. In the multivariate logistic regression analysis, hypertension and CrCP remained independent determinants of CSVD burden (Table 2). After adjusting for age, sex, and significant (*P* < 0.05) variables from the univariate analyses in Model 1, only hypertension and CrCP were independently associated with CSVD burden. After additionally adjusting for the risk factors or hemodynamics, in Model 2 or 3, hypertension and CrCP remained independent determinants. Furthermore, we adjusted for all variables above, including age, sex, risk factors, hemodynamics, and significant variables from the univariate analyses in Model 4, hypertension [OR (95% CI): 1.06 (1.00–1.63); *P* = 0.020] and CrCP [OR (95% CI): 1.41 (1.17–1.71); *P* < 0.001] were still the independent determinants of CSVD burden.

**Table 2:** Determinants of CSVD burden in multivariate models^a^.

**Variables**	**OR (95% CI)**	***p***
**Model 1**		
Hypertension	1.13 (1.03–1.64)	0.012
CrCP	1.30 (1.14–1.49)	<0.001
**Model 2**		
Hypertension	1.14 (1.02–1.98)	0.047
CrCP	1.38 (1.16–1.66)	<0.001
**Model 3**		
Hypertension	1.08 (1.01–1.47)	0.005
CrCP	1.29 (1.13–1.47)	<0.001
**Model 4**		
Hypertension	1.06 (1.00–1.63)	0.020
CrCP	1.41 (1.17–1.71)	<0.001

OR, odds ratio; CI, confidence interval; CrCP, critical closing pressure; CSVD, cerebral small vessel disease.

^a^Model 1 adjusted for age, sex, and significant (P < 0.05) variables from the univariate analyses (Including age, hypertension, systolic blood pressure, diastolic blood pressure, mean blood pressure, pulse pressure, and CrCP); Model 2 adjusted for age, sex, vascular risk factors (Including hypertension, diabetes mellitus, coronary artery disease, current or recent smoking history, alcohol consumption history, BMI, triglyceride, cholesterol, low density lipoprotein, and Glucose) and significant variables from the univariate analyses; Model 3 adjusted for age, sex, hemodynamics (Including systolic blood pressure, diastolic blood pressure, mean blood pressure, pulse pressure, PSV, MFV, PI, and CrCP) and significant variables from the univariate analyses; and Model 4 adjusted for age, sex, vascular risk factors, hemodynamics and significant variables from the univariate analyses.

### CrCP, PI and CSVD burden

**Figure 1:**
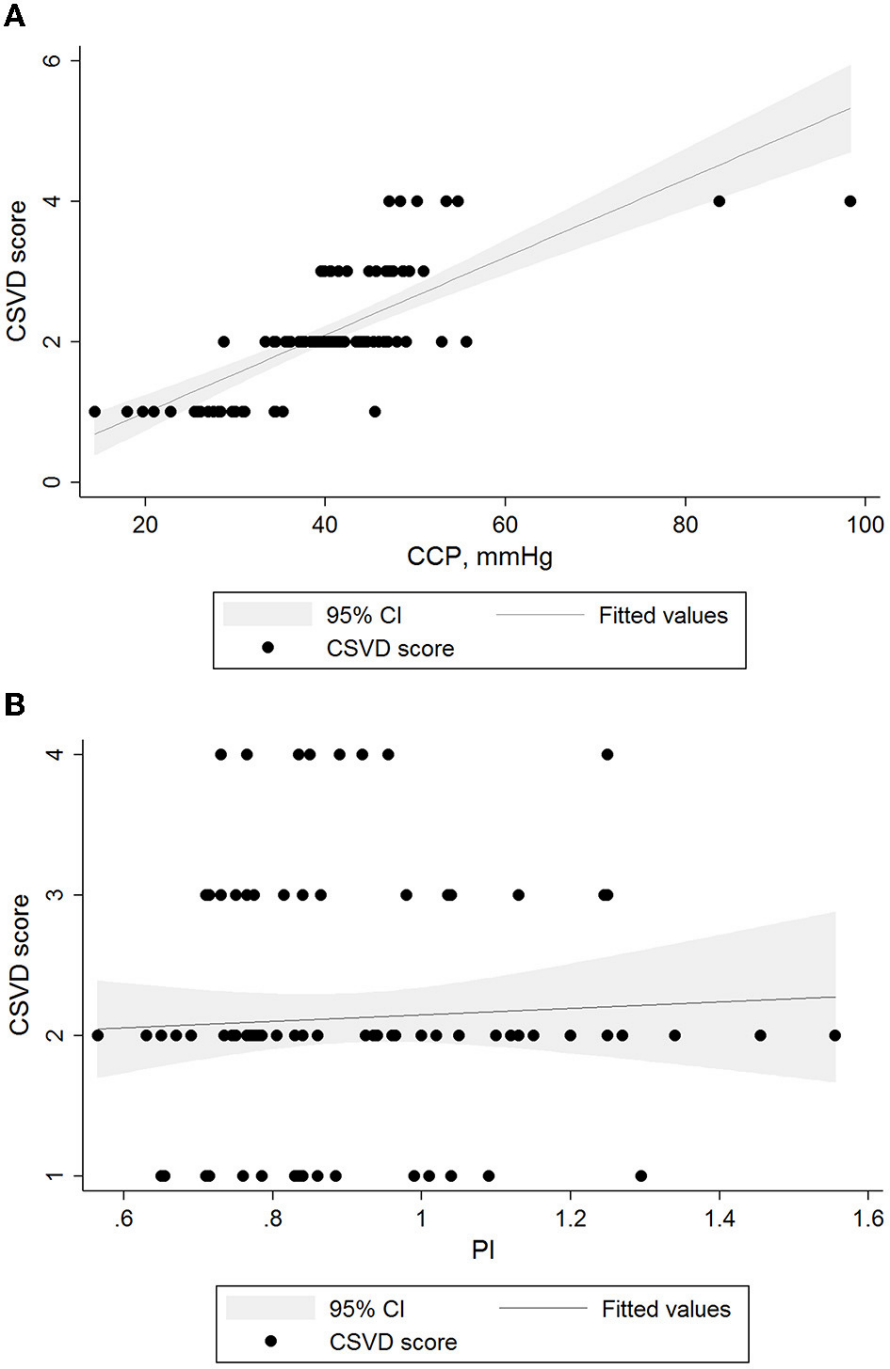
Relationships between levels of hemodynamic parameters and CSVD burden. Black lines and gray shadows represent the estimated probability and 95% CIs for the CSVD scores at the level of methods on the basis of the generalized additive model. The x axis is limited from the 5th to the 95th percentile of the level of the hemodynamic parameter. **(A)** CSVD scores and CrCP β = 0.05 (95% CI 0.04 to 0.06, *p* < 0.01). **(B)** CSVD scores and PI β = −0.76 (95% CI −1.63 to 0.12, *p* = 0.091). CI, confidence interval; CSVD, cerebral small vessel disease; PI, pulsatility index.

In univariate linear regression analysis (Table 3), CVR and CrCP were associated with CSVD burden (all *P* < 0.05). After adjusting for age, sex, and significant (*P* < 0.05) variables from the univariate analyses (Table 3), we found that CSVD burden remained associated with CrCP [β (95% CI): 0.05 (0.04–0.06); *P* < 0.001], but did not have the similar relationship with PI [β (95% CI): −0.29 (−1.00 to 0.42); *P* = 0.416]. On visual inspection, we also found that only CrCP correlated with the CSVD score in the two hemodynamic parameters (Figure 1).

**Table 3:** CSVD burden association with demographics and baseline characteristics.

**Characteristics**	**Univariate**		**Multivariate** ^**a**^	
	β **(95% CI)**	***p***	β **(95% CI)**	***p***
Male	−0.39 (−0.79 to 0.04)	0.053	−0.10 (−0.39 to 0.18)	0.475
Age, y	0.03 (0.01 to 0.05)	<0.001	0.01 (−0.01 to 0.02)	0.340
Hypertension	0.30 (−0.05 to 0.64)	0.090	0.22 (−0.04 to 0.48)	0.096
Diabetes mellitus	0.011 (−0.46 to 0.49)	0.962	−0.14 (−0.49 to 0.20)	0.405
Coronary artery disease	0.33 (−0.18 to 0.85)	0.201	0.25 (−0.12 to 0.62)	0.182
Current or recent smoking history	0.08 (−0.30 to 0.46)	0.686	−0.06 (−0.34 to 0.22)	0.686
Alcohol consumption history	−0.03 (−0.59 to 0.54)	0.926	0.10 (−0.32 to 0.51)	0.654
BMI	−0.04 (−0.06 to 0.05)	0.873	−0.03 (−0.07 to 0.01)	0.115
Triglyceride	−0.08 (−0.28 to 0.12)	0.419	−0.12 (−0.26 to 0.02)	0.097
Cholesterol	−0.03 (−0.16 to 0.11)	0.704	−0.01 (−0.11 to 0.08)	0.767
Low density lipoprotein	−0.09 (−0.26 to 0.08)	0.295	−0.04 (−0.16 to 0.08)	0.497
Glucose	−0.08 (−0.20 to 0.04)	0.170	−0.06 (−0.14 to 0.02)	0.149
Systolic blood pressure	0.01 (0.01 to 0.02)	<0.001	0.00 (0.00 to 0.01)	0.394
Diastolic blood pressure	0.02 (0.01 to 0.03)	<0.001	0.00 (−0.01 to 0.02)	0.866
Mean blood pressure	0.02 (0.01 to 0.03)	<0.001	0.00 (−0.02 to 0.02)	0.866
Pulse pressure	0.02 (0.01 to 0.03)	<0.001	0.00 (−0.02 to 0.01)	0.866
PSV	−0.01 (−0.01 to 0.01)	0.874	0.00 (−0.01 to 0.00)	0.137
MFV	−0.03 (−0.01 to 0.01)	0.604	−0.01 (−0.01 to 0.00)	0.203
PI	0.23 (−0.65 to 1.12)	0.604	−0.29 (−1.00 to 0.42)	0.416
CrCP	0.06 (0.05 to 0.07)	<0.001	0.05 (0.04 to 0.06)	<0.001

CrCP, critical closing pressure; CI, confidence interval; BMI, body mass index; PSV, peak systolic velocity; MFV, mean flow velocity; PI, pulsatility index; WMH, white matter hyperintensities; PVS, Perivascular spaces.

^a^All multivariable regression models adjusted for age, sex, systolic BP and CrCP.

**Figure 2:**
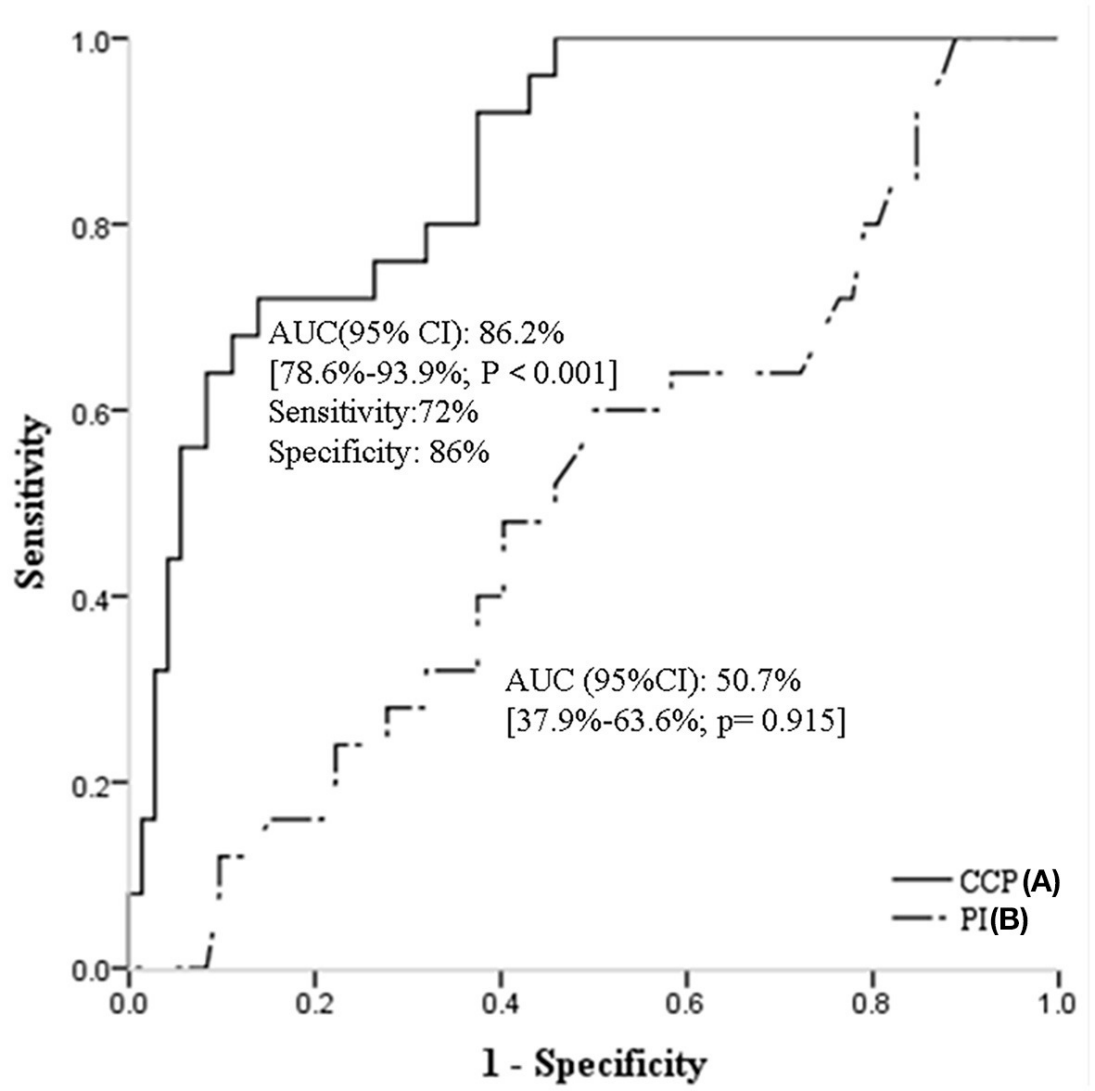
Receiver operating characteristic curves of CrCP **(A)**, and PI **(B)** for predicting CSVD burden. CSVD, cerebral small vessel disease; CrCP, critical closing pressure; PI, pulsatility index.

In ROC analysis, when the continuous value of CrCP was considered as a predictor of CSVD burden (Figure 2A), the AUC was 86.2% (95% CI, 78.6%-93.9%; *P* < 0.001). The sensitivity was 72%, and the specificity was 86%. However, the ROC curves of PI [AUC (95%CI): 50.7% (37.9–63.6%; *p* = 0.915)] did not present good predictive values for CSVD burden (Figure 2B).

### CrCP and patient characteristics

In univariate linear regression analysis (Table 4), Age, systolic and diastolic BP, MBP and Pulse pressure were associated with CrCP (all *P* < 0.05). After adjusting for age, sex, and significant (*P* < 0.05) variables from the univariate analyses, age [β (95% CI): 0.27 (0.06–0.47); *P* = 0.012], BMI [β (95% CI): 0.61 (0.00–1.22); *P* = 0.049] and systolic BP[β (95% CI): 0.16 (0.09–0.23); *P* < 0.001] remained associated with CrCP.

**Table 4:** CrCP association with demographics and baseline characteristics.

**Characteristics**	**Univariate**		**Multivariate** ^**a**^	
	β **(95% CI)**	***p***	β **(95% CI)**	***p***
Male	−4.79 (−10.03 to 0.47)	0.074	−2.30 (−6.86 to 2.27)	0.320
Age, y	0.44 (0.23 to 0.65)	<0.001	0.27 (0.06 to 0.47)	0.012
Hypertension	0.67 (−3.95 to 5.28)	0.774	−4.00 (−8.10 to 0.10)	0.056
Diabetes mellitus	1.47 (−4.81 to 7.75)	0.643	−3.36 (−8.86 to 2.14)	0.228
Coronary artery disease	1.05 (−5.85 to 7.94)	0.764	−1.30 (−7.30 to 4.71)	0.669
Current or recent smoking history	1.52 (−3.49 to 6.52)	0.549	−0.25 (−4.81 to 4.31)	0.914
Alcohol consumption history	−1.79 (−9.25 to 5.67)	0.635	0.65 (−6.02 to 7.32)	0.847
BMI	0.55 (−0.17 to 1.26)	0.132	0.61 (0.00 to 1.22)	0.049
Triglyceride	0.91 (−1.70 to 3.52)	0.490	1.78 (−0.44 to 4.00)	0.114
Cholesterol	0.05 (−1.72 to 1.81)	0.052	0.45 (−1.07 to 1.97)	0.560
Low density lipoprotein	−0.61 (−2.90 to 1.68)	0.596	0.20 (−1.77 to 2.18)	0.839
Glucose	−0.45 (−2.00 to 1.10)	0.567	−0.66 (−1.97 to 0.65)	0.322
Systolic blood pressure	0.20 (0.13 to 0.27)	<0.001	0.16 (0.09 to 0.23)	<0.001
Diastolic blood pressure	0.32 (0.18 to 0.46)	<0.001	0.09 (−0.14 to 0.32)	0.461
Mean blood pressure	0.30 (0.19 to 0.40)	<0.001	0.13 (−0.22 to 0.47)	0.461
Pulse pressure	0.26 (0.15 to 0.37)	<0.001	−0.09 (−0.32 to 0.14)	0.461
PSV	0.03 (−0.07 to 0.12)	0.574	−0.07 (−0.15 to 0.02)	0.125
MFV	0.02 (−0.14 to 0.17)	0.843	−0.06 (−0.19 to 0.07)	0.363
PI	4.26 (−7.46 to 15.98)	0.472	−9.70 (−20.88 to 1.48)	0.088

CrCP, critical closing pressure; CI, confidence interval; BMI, body mass index; PSV, peak systolic velocity; MFV, mean flow velocity; PI, pulsatility index; CVR, cerebrovascular reactivity; WMH, white matter hyperintensities; PVS, Perivascular spaces.

^a^All multivariable linear regression models adjusted for age, sex and systolic BP.

## Discussion

This is the first to systematically evaluate the relationship between cerebrovascular tone and CSVD burden. The results of recent studies demonstrated that cerebrovascular hemodynamics may play important roles in the pathophysiological mechanism of CSVD, but cerebrovascular tone was not involved (4, 5, 7). As we know, CrCP equals the sum of ICP and cerebrovascular tone and is confirmed mainly reflecting the behavior of cerebrovascular tone rather than ICP (13, 14, 17). Since CrCP is widely recognized as the parameter for estimating the changes of cerebrovascular tone (11, 12, 17–20), we used CrCP to estimate the changes of cerebrovascular tone in this study. In addition, we recruited SVO patients (NIHSS and mRS scores both <3) as participants from our AIS database to ensure relevance to patients who are commonly affected by CSVD. Their ICPs are relatively constant, and their CrCP can better reflect the state of cerebrovascular tone in the present study.

The main finding of this study is that CrCP is significantly correlated with CSVD burden. We demonstrated that CrCP was an independent determinant of CSVD burden in the multivariate logistic regression analysis, after adjusting for age, sex, and significant variables from the univariate analyses in four models. Furthermore, we observed that CrCP associated with CSVD burden in the multivariable linear regression analysis, and they also showed a similar relationship in the generalized additive regression model on visual inspection. Importantly, we found that CrCP could be considered as a predictor of CSVD burden in ROC analysis; the sensitivity was 72%, and the specificity was 86%. Numerous studies have discussed the association between cerebral hemodynamic parameters with CSVD (1, 4–7, 26–29). However, reliable data concerning relations between CrCP and CSVD are lacking. In the present study, the gap was filled and CrCP was shown as a new hemodynamic marker of CSVD burden. CrCP is defined as the level of ABP at which small arteries in the brain close and cerebral blood flow ceases, and mainly represents the cerebrovascular tone. Previous study has demonstrated that CrCP determines the effective downstream pressure of the cerebral circulation (17). Cerebral perfusion pressure (CPP), a major determinant of cerebral blood flow, is calculated from the difference between mean arterial pressure and the downstream pressure of the cerebral circulation (17). Therefore, not only is CrCP correlated with CPP (9, 10, 14, 17, 30), but CrCP further affects the CBF of cerebral microvessels. Numerous studies have confirmed that CBF is a crucial factor in the pathogenesis of CSVD (1, 6, 28, 29), Thus, this may be a pathway for CrCP to participate in the pathogenesis of CSVD. In addition, we also found that higher CrCP values in this study were significantly correlated with older age, higher BMI and systolic BP, which are the traditional risk factors of CSVD. The interaction between CrCP and traditional risk factors of CSVD may be another possible way for CrCP to participate in the pathogenesis of CSVD. However, this hypothesis needs to be verified in future studies.

We did not find associations of PI with CSVD burden. Associations between PI and WMH have been discussed in the previous studies, but the results were inconsistent (7, 31). To explore the relationship between PI and CSVD burden in the present study, we performed a variety of statistical analysis methods such as multivariate logistic regression, linear regression analysis and ROC analysis, but the results were negative. Furthermore, we established a fractional polynomial plot with 95% CIs for the CSVD scores according to the level of PI based on the generalized additive regression model. Likewise, we did not observe the associations on visual inspection. This may be due to lack of statistical power, and may also be related to the difference between the whole and the part of CSVD imaging features.

The present study has both strengths and limitations. The strengths include the following: First, this study systematically verified associations between CrCP and CSVD burden. The present results demonstrate that CrCP is an independent determinant and predictor of CSVD burden, which will provide reliable data for exploring the role of cerebrovascular tone in the pathogenesis of CSVD in future. Second, all participants were recruited from our AIS database and were subjected to careful selection using validated scales and multimodal brain MR. Third, the CrCP was measured by experienced operators with intensive training using gold standard techniques. However, the present study was performed at a single center and included a population with a single ethnicity. Meanwhile, we recruited SVO patients as participants from our AIS database in the present study, which would ignore the patients with chronic cerebrovascular disease and lead to a selection bias. Furthermore, CrCP was estimated according to Rune Aaslid's method using TCD. This classic method is burdened with assumptions of linearity between ABP and CBF, which may cause underestimation of CrCP. In addition, only 97 patients with SVO were enrolled as participants in this study, the sample size limited the statistical power, and any result should be considered for clinical plausibility and need to be further verified in future studies with larger sample size.

In conclusion, CrCP representing cerebrovascular tension is an independent determinant and predictor of CSVD burden. It was significantly correlated with age, BMI and systolic blood pressure. These results provide new insights in the mechanism of CSVD development.

## Data availability statement

The original contributions presented in the study are included in the article/Supplementary material, further inquiries can be directed to the corresponding authors.

## Ethics statement

The studies involving human participants were reviewed and approved by the Ethics Committee of the Second Affiliated Hospital of Guangzhou Medical University approved the study protocol. The patients/participants provided their written informed consent to participate in this study.

## Author contributions

XF: design, statistical analysis, and writing. WZ: hemodynamic data acquisition and analysis and data collection. XL: design and data collection. HL and YZ: hemodynamic data acquisition and analysis. QG: design and guiding. All authors contributed to the article and approved the submitted version.
