# Supplementary material for: Critical closing pressure as a new hemodynamic marker of cerebral small vessel diseases burden
Source: Front Neurol. 2023 Mar 21;14:1091075. doi: 10.3389/fneur.2023.1091075 (PMC10071665; doi:10.3389/fneur.2023.1091075)
Supplement: Supplementary file 1 [file Table_1.DOC]

**Supplementary Information**

Table1. Univariate analysis of the determinants of CSVD burden.

| Characteristics | OR (95% CI) | P value |
| --- | --- | --- |
| Male | 1.88(0.57-6.19) | 0.298 |
| Age, y | 1.07(1.02-1.12) | 0.009 |
| Hypertension | 1.26(1.10-1.78) | 0.016 |
| Diabetes mellitus | 0.95(0.27-3.30) | 0.931 |
| Coronary artery disease | 0.43(0.12-1.51) | 0.187 |
| Current or recent smoking history | 1.39(0.49-3.97) | 0.534 |
| Alcohol consumption history | 0.79(0.19-3.32) | 0.747 |
| BMI | 0.97(0.84-1.12) | 0.658 |
| Triglyceride | 0.83(0.45-1.51) | 0.532 |
| Cholesterol | 0.86(0.60-1.23) | 0.405 |
| Low density lipoprotein | 0.71(0.44-1.15) | 0.168 |
| Glucose | 0.72(0.45-1.16) | 0.718 |
| Systolic blood pressure | 1.02(1.01-1.04) | 0.013 |
| Diastolic blood pressure | 1.04(1.00-1.08) | 0.033 |
| Mean blood pressure | 1.04(1.01-1.06) | 0.016 |
| Pulse pressure | 1.03(1.00-1.05) | 0.031 |
| PSV | 0.99(0.97-1.01) | 0.547 |
| MFV | 0.99(0.95-1.02) | 0.432 |
| PI | 0.94(0.09-9.99) | 0.961 |
| CCP | 1.27(1.13-1.42) | ＜0.001 |

Abbreviations: OR = odds ratio; CI = confidence interval; BMI = body mass index; IQR = Interquartile Range; PSV = peak systolic velocity; MFV = mean flow velocity; PI = pulsatility index; CCP = critical closing pressure; CSVD = cerebral small vessel disease.
